# Energy supply and influencing factors of mountain marathon runners from Baiyin marathon accident in China

**DOI:** 10.1038/s41598-022-12403-1

**Published:** 2022-05-17

**Authors:** Jichao Sun

**Affiliations:** School of Water Resource & Environment, China University of Geosciences, Beijing, 100083 China

**Keywords:** Metabolism, Natural hazards

## Abstract

High temperature impacts the performance of marathon athletes, and hypothermia harms athletes. Twenty-one runners died, and eight were injured in the China Baiyin marathon on May 22, 2021. It’s a typical human life test. The energy equations are combined with the maximum energy supply of Chinese male athletes to study this accident. We analyze the human body’s route slope, travel speed, and heat dissipation under low temperatures in this marathon. The study shows that the large slope and long-distance of CP2 to CP3 section and the low temperature during the competition are the main reasons for the accident. The method of quantifying the slope and temperature and calculating the percentage of athletes’ physical consumption proposed in this paper can evaluate the route design of field marathons. We suggest that the physical energy consumption ratio of 90%, i.e. 315 cal/min/kg, should be taken as the maximum energy supply for Chinese male marathon runners. Dangerous risk zones for wind speed and temperature on dangerous path sections are also formulated for athletes to make their assessments. This paper’s theories and methods can effectively help design the marathon route and determine the race time.

## Introduction

Sports is an activity developed to meet the needs of human production, military, and health^1^. Physical fitness is the premise to maintain and complete the human body’s essential life activities and physical skills. Marathon runs for a long time and consumes much energy. The performance of athletes is closely related to physical fitness^2^. Physical performance is also affected by the surrounding environment, including temperature, humidity, road slope, road friction, and so on^3, 4^. The relationship between human energy expenditure and the external environment needs to be studied to assess the individual strength of marathon runners. The war between Russia and Ukraine is going on, and now ordinary Ukrainian residents are also participating in the war. These people lack military training and actual combat experience and have limited combat power to participate in the war. Obtaining the maximum energy supply of the human body also helps assess the combat effectiveness of a soldier^5^ and ordinary people to participate in war.

For many years, sports researchers around the world have been conducting similar studies, including pre-competition hypothermia, post-competition hypothermia, and pre-competition and post-competition warming studies^6^ at the cold Winter Olympics, as well as various Oxygen inhalation, high-intensity training at high altitudes^7–10^, etc., these studies have achieved positive results.

High temperature impacts the performance of marathon athletes, and hypothermia harms athletes^11, 12^. The research on the limited energy supply means the final limit of the human body’s skills, and it is the research at the expense of the human body’s death. When evaluating the energy supply of marathon runners or ordinary people, especially their limited energy supply, it is impossible to carry out limit tests to ensure personnel safety. At the same time, there is still a lack of published literature on this kind of extreme energy supply research. From some marathon fatalities, we can get some trial-like results. Analyzing these marathon accidents, one can obtain data on the extreme energy supply of the personnel. Although these tests are not obtained from actual tests, these data are relatively close to the limits of the human body and are very precious. At the same time, such data is essential to the military and sports. After obtaining such data, it is possible to design better the marathon running route and guide the athletes to conduct self-assessments whether they can refer to relevant competitions, which will have positive significance and value. It is also a good guide for evaluating ordinary people who participated in the war.

To increase the influence and popularity of the city, local governments in China have held marathons one after another and invited famous runners from all over the world to participate, which has indeed boosted the local economy in China. In 2018 and 2019, there were 278, 330, and 27, 61 registrations for Category A and Category B events of the Chinese Athletics Association respectively^13^. As of March 19, 2022, the running competitions held in China include various walks, half marathons, 50 km, 30 km, 28 km, 21 km, 16 km, 15 km, 10 km, 8, 8 km, 3.8 km, and so on to 2625 times^14^.

But there have been very individual race accidents, such as the marathon accident in Baiyin, Gansu of China^15^. On May 22, 2021, the fourth Yellow River Shilin Mountain Marathon 100 km cross-country race (Abbreviated as Baiyin marathon) was held in Jingtai County, Baiyin City of China. 172 participants took part in the 100-km cross-country race. During the race, public safety responsibility events occurred due to sudden cooling, precipitation, and strong wind, resulting in the death of 21 participants and the injury of 8^15^. China’s State Sports Administration issued a notice on May 28 to comprehensively ‘one-on-one’ check road running (including marathon, half marathon, 10 km, 5 km) and suspend the competitions that do not meet the requirements. On June 25, the government arrested five officials and dismissed, removed, demoted, warned, and demerit recorded twenty-seven leaders^15^.

**Figure 1:**
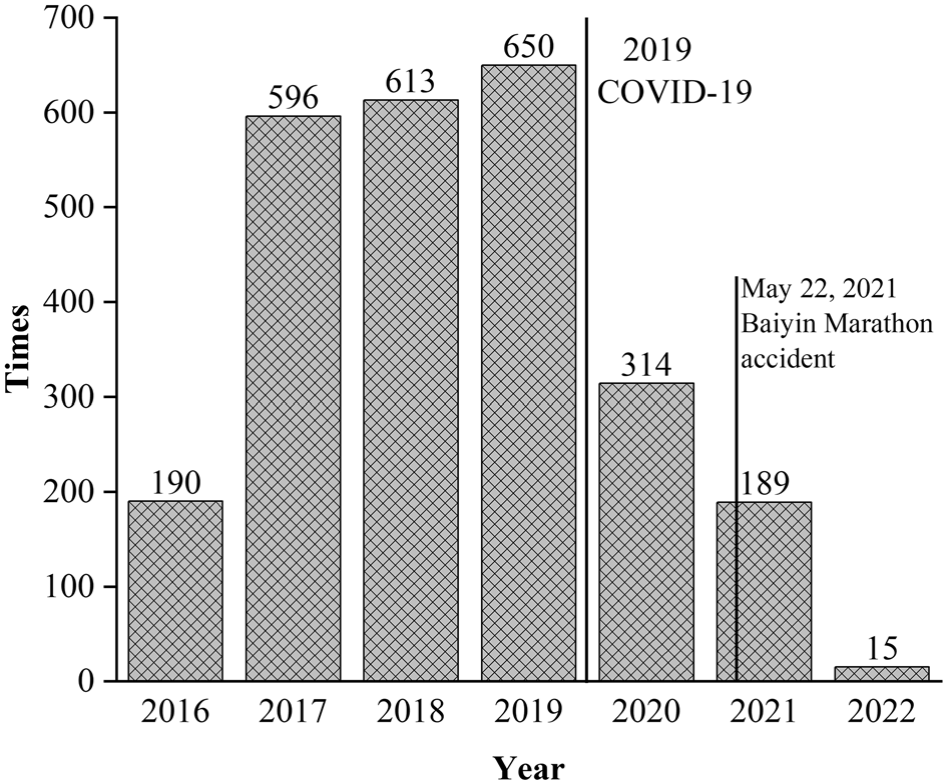
Many marathons held in China.

It can be seen from Fig. 1 that due to COVID-19, the number of marathons has significantly been reduced since 2020. Due to the Baiyin marathon accident in 2021, the State Sport General Administration of China immediately stopped many marathons, so the number of races in 2021 was reduced. Data for 2022 is not final yet, and complete data will be available in 2023.

On the one hand, marathon accidents are not good for the development of marathon sports, and on the other hand, it is also bad for the health of the athletes participating in the competition. The high temperature in this kind of competition, like a marathon, or long-distance races, has attracted the attention of athletes and relevant media^16^. However, low temperature also does great harm to athletes^17^. There are many reasons for the accident in the Baiyin marathon. However, the low temperature and the energy supply of the athletes’ bodies are less than the energy dissipation is one of the main reasons.

Because of this severe sports accident, it is necessary to conduct scientific research, analyze the causes of the accident, and avoid such accidents. Local governments hold various competitions to promote tourism development, stimulate the economy, and improve local popularity. Ignoring the potential hazards is an important reason why many Gansu province officials were held accountable for an ultramarathon tragedy^18^. At the same time, the Baiyin marathon accident also has several adverse natural conditions: high altitude, large slope, and low temperature.

This paper studies the route slope and temperature from the human body’s heat dissipation and energy supply to reveal the cause of the accident.

In this accident, the athlete’s death has already shown that the energy supply has reached its limit. It is simply a life experiment of the human body, which is very cruel. Therefore, it is necessary to study the energy supply limit of athletes in this competition through this marathon accident and analyze the cause of the accident from the human energy supply limit. Therefore, the main goal of this paper is to study and obtain the human body's limited supply of energy for remote mobilization and combine the slope of the track and the temperature of the environment to study and analyze the setting of the way.

Avoid recurrence of such accidents through research. At the same time, we also put forward some suggestions for athletes to participate in similar competitions. We set up a self-assessment chart for whether sports can participate based on temperature, slope, and speed during the competition. It is hoped that casualties in future competitions can be avoided through this accident analysis.

## Methods

### The elevation and landform of the Biayin marathon

**Figure 2:**
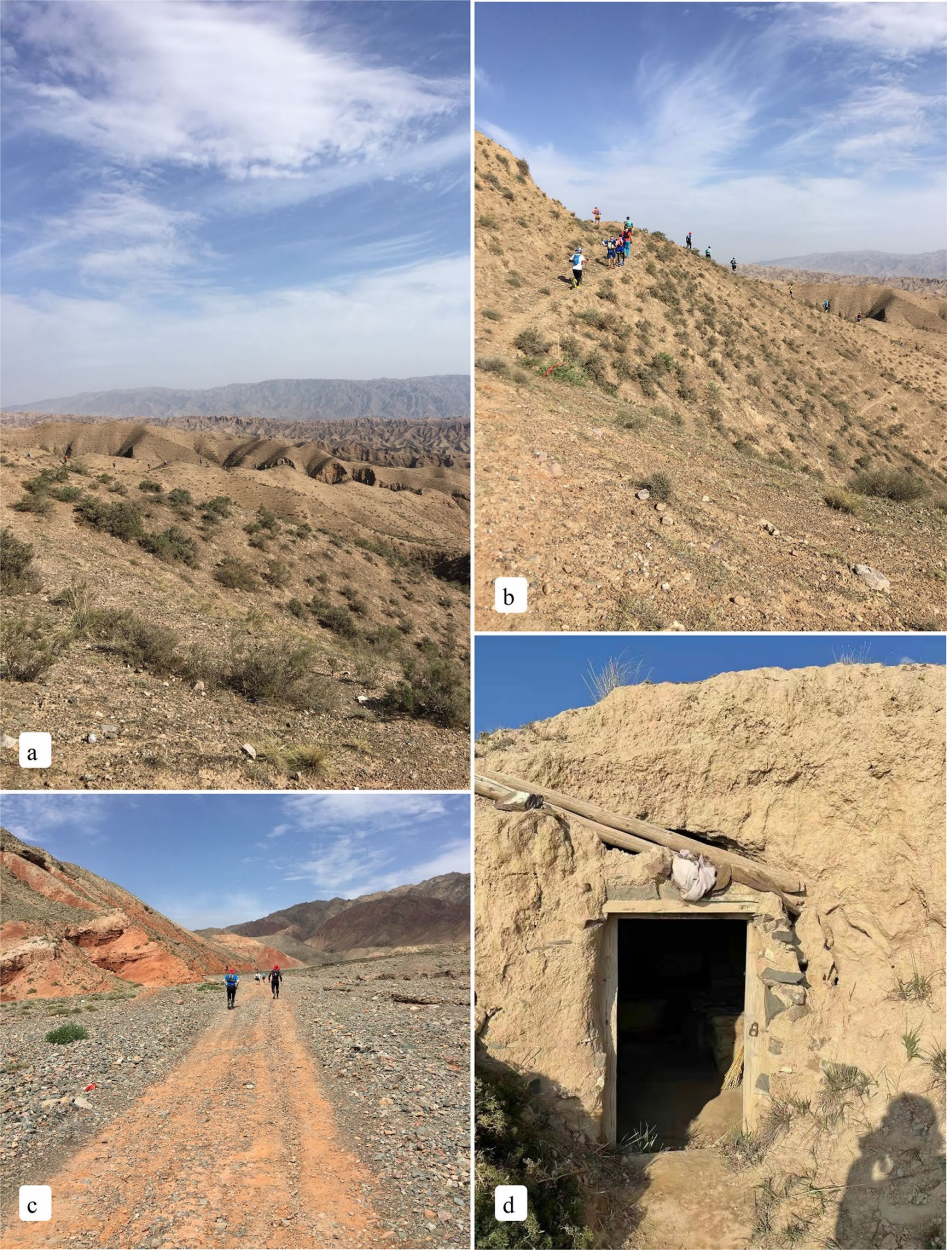
Baiyin marathon photos of pre-race, disaster, and rescue. (**a**–**c**) Baiyin marathon on May 20, 2018. (**d**) cave-dwelling, some runners were warming themselves by the fire on May 22, 2021.

**Figure 3:**
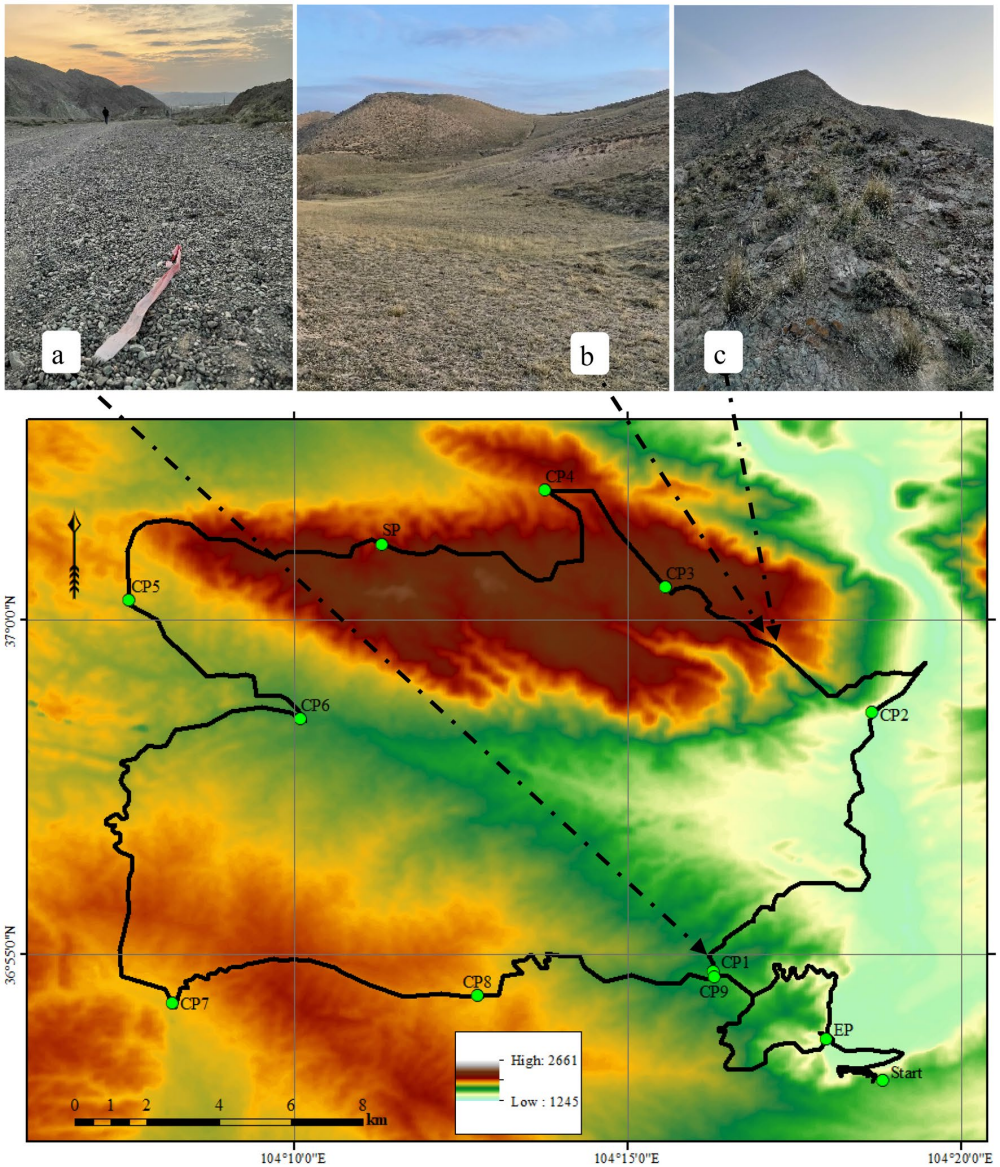
Marathon route and start, check, supply and endpoint and pictures along the route. (**a**) Photo of the race route on CP1. (**b,c**) photos of the race route on CP2–CP3. ‘Start’ is the starting point, CP1–CP9 is the checkpoint, SP is the supply point, and EP is the endpoint.

Baiyin marathon in Gansu of China was held four times in 2018 (Fig. 2a–c), 2019, 2020, and 2021. The first three sessions went well, and an accident occurred in the fourth session (some runners were warming themselves in the cave-dwelling, Fig. 2d). According to the race route of the Baiyin marathon, the elevation map in the race route area is drawn, and a map of vegetation along the way is attached (as shown in Fig. 3).

Baiyin city is mountainous primarily, and a broad valley plain coexists. The northern part is an alluvial diluvial inclined plain, the central part is a low hill, and the southern part is loess beam, replat, and tableland landform.

The climate of Baiyin city is a transition zone from a middle temperate semi-arid zone to an arid zone in China. The annual average temperature is 6–9 ℃, and the yearly rainfall is 180–450 mm, mainly in July, August, and September, accounting for more than 60% of the annual precipitation. It belongs to the northwest margin of the southeast monsoon climate, and the yearly evaporation reaches 1500–1600 mm, 4.5 times the average precipitation. Jingtai County in the north has maximum annual evaporation of 3390 mm. Baiyin has four seasons with plenty of sunshine, no hot summer, and no cold winter.

The topographic features of Baiyin City are mainly bedrock mountains (as shown in Figs. 2c, 3a) and intermountain basins. The horizontal distribution of vegetation in the city gradually transitions from south to north to grassland deserts (as shown in Fig. 3b), and the transition between zones is not apparent. The tectonic structure in the northwest belongs to the eastward extension of the Qilian Mountains, with bare ground bedrock (as shown in Fig. 2c) and natural vegetation on the shady slopes (as shown in Figs. 2a,b, 3c). The southeast is dominated by loess beam, replat and tableland landform, rivers, flats, and valleys. The tectonic structure belongs to the Longzhong Basin. Except for a few bedrock mountains, the ground is covered by loess (Fig. 2d).

### Energy consumption at a particular slope and speed

Marathon runner’s energy consumption includes heat dissipation and kinetic energy consumption of the human body. The athletes need to supply more energy to meet heat dissipation and kinetic energy consumption when the temperature is lower, the travel speed is faster, and the road is steeper. The athlete can complete sports at low temperatures, large slopes, and high speeds only when the speed and intensity of the energy supplied by the human body meet these energy requirements.

At the general running speed, the prediction formula of human exercise energy consumption^19, 20^ is:

1 $$M = 1.5W + 2.0(W + L)\left( \frac{L}{W} \right)^{2} + \eta (W + L)[1.5V^{2} + 0.35VG],$$

where *M* is the energy consumption rate of the human body (*w*), *W* is the body weight (kg), *L* is the bear load (kg), *V* is the running speed (m/s), *G* is the slope (%), *η* is the surface condition coefficient, *η* = 1 in the case of horizontal hard road and *η* = 2 on loose sand. Figures 2a–c and 3a–c, *η* = 1.2.

People move slower on a 12° gradient slope uphill and downhill than on a flat road^21^. When going downhill, the energy consumption decreases^22^, but with the increase of the descending slope, the energy consumption also increases to improve the friction and reduce the risk of falling^23^. But the increase is not as significant as the upslope walking. This paper selects the positive slope for calculation.

### Human body surface heat dissipation

Baiyin Marathon runners are not equipped for the cold, wearing simple clothes or directly exposed^24–26^. The heat dissipation is,

2 $$Wci = (10.45 + 10\sqrt F - F) \times \left( {33 - T} \right),$$

where *Wci* is the air-cooling index (kcal/m^2^/h), *F* is the wind speed (m/s), *T* is the air temperature (℃). It represents the heat dissipation rate of the human body surface with a skin temperature of 33 ℃.

### The maximum energy supply of Chinese runners

Accurate measurement of lipid or carbohydrate metabolism during exercise is also complex. Marathon running is fueled principally by the oxidation of intramuscular glycogen and lipids to a lesser extent^27, 28^. Therefore, there is no need to distinguish between carbohydrate and lipid metabolism and their percentages.

At the same temperature, the energy consumption positively correlates with the maximum oxygen uptake^29^. According to the test results^30, 31^, perform linear formula fitting at the same temperature to obtain the energy consumption at 100% VO_2max_, namely the maximum energy supply rate. Table 1 lists the data. In reference^29^, the performance at different temperatures shows a similar and linear relationship.

**Table 1:** Reference data of human of Chinese male athletes’ energy supply.

Intensity (% of VO_2max_)	Total energy consumption (cal/min/kg)	Temperature (°C)	References
65%VO_2max_	240.0	12	^30^
80%VO_2max_	290.0		
100%VO_2max_	356.7		This research
40%VO_2max_	134.8	23	^31^
60%VO_2max_	185.4		
80%VO_2max_	239.8		
100%VO_2max_	291.7		This research
40%VO_2max_	126.6	33	^31^
60%VO_2max_	188.3		
80%VO_2max_	236.7		
100%VO_2max_	294.0		This research

Rising and falling external temperatures increase the body’s energy expenditure (Table 1).

The linear relationship between the total energy consumption of Chinese male athletes at different temperatures of 100%VO_2max_ and temperature is not significant. On the contrary, the total energy consumption at different temperatures remains stable^32^. It isn’t easy to obtain data on the maximum energy supply at different temperatures. Therefore, this paper takes the full energy supply as a fixed value. The energy supply range of Chinese male athletes is 291.7–356.7 cal/min/kg. Therefore, this paper considers that Chinese male athletes' maximum energy supply rate is 350 cal/min/kg.

### Other data

Select terrain data from ASTERGDEMv2.0, land DEM, with an accuracy of about 30 m, and the download address is https://earthexplorer.usgs.gov/. Figure 3 is generated by ArcGIS V10.3.

Liang Jing, China’s leading marathon runner who died in the accident, ran at 100 km for 7 h in previous competitions at a speed of 4 m/s. This research takes the value of 1.5 m/s according to the running speed of ordinary athletes. The weight of male athletes is 70 kg, the wind speed is 0, the height is 170 cm, the body surface area is 1.82 m^2^, and the temperature comes from Baiyin meteorological observation station.

**Figure 4:**
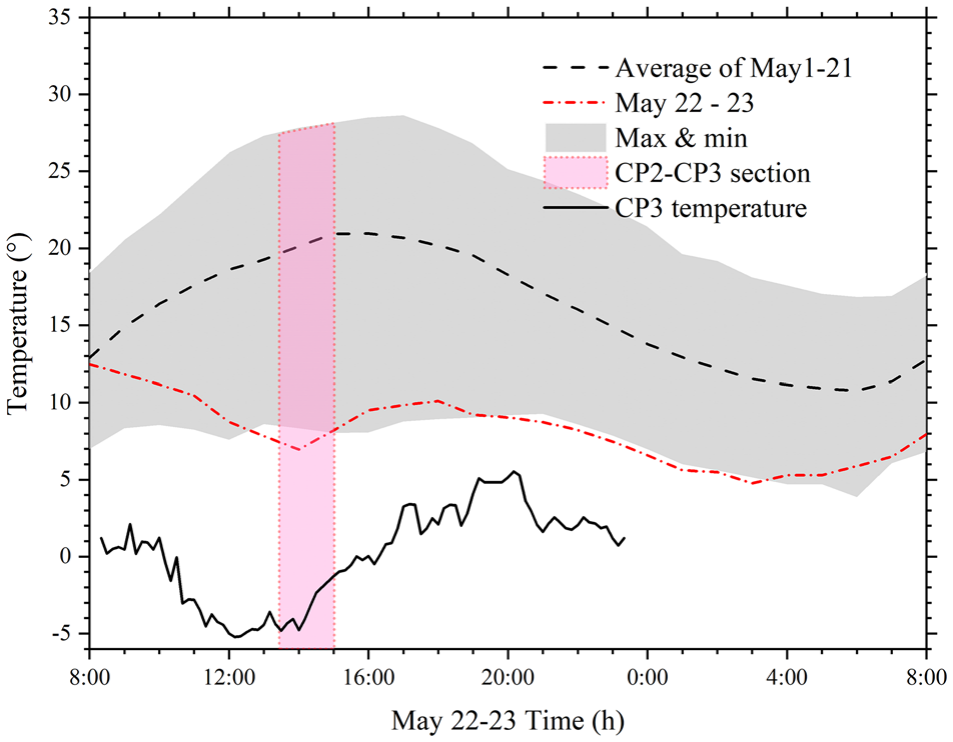
The temperature of Baiyin and CP3 on race day and the average temperature, maximum and minimum temperature of 21 days before the competition, Baiyin Weather Station.

Reference^15^ provides the temperature data of the day at point CP3. Baiyin Weather Station and Reference^33^ offer the average temperature, maximum and minimum temperature of 21 days before the competition. Figure 4 lists the temperature curves^15^.

## Results

**Figure 5:**
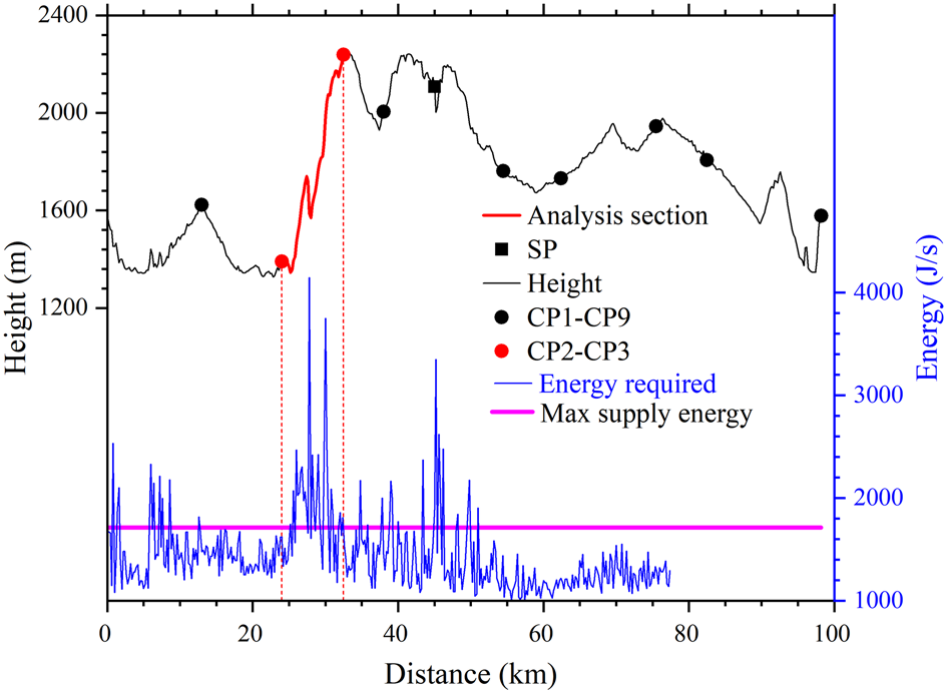
Marathon route altitude, maximum energy supply, and energy demand curve, replenishment points, and check-in points. Calculate the energy required according to the actual temperature at CP3. Since there is no temperature data later, the following data is vacant.

According to Eqs. (1) and (2) calculate the total energy required by running energy consumption and surface heat dissipation along the elevation of the running route. And the energy supply rate is 350 cal/min/kg (1715 J/s, max supply energy) according to the maximum oxygen uptake. Figure 5 lists the results.

The slope increases significantly in the CP2–CP3 section (24–32.5 km), and the required energy rises. According to the speed of 1.5 m/s, runners run in this section from 13:26 to 15:01. At this time, the temperature in Baiyin is 6.9–8.2 ℃. In Fig. 5, many parts of the blue curve of CP2–CP3 section are more significant than the pink curve, indicating that the energy demand is greater than the energy supply. The external performance is that the athletes gradually lose temperature and enter a dangerous state. In the section before CP1 (5–10 km), there is also a part where the energy demand is greater than the energy supply. Still, this section is at the initial running stage, and the gap between energy demand and energy supply is not too large. Athletes can well avoid temperature loss in this section by unconscious deceleration. The energy demand is greater than the energy supply from CP4 to SP and SP to CP5, but the two sections are mainly the downhill section. Since calculating in this paper is based on the absolute value of the slope, athletes can slow down and travel downhill slowly. At the same time, the distance is not long, and the actual energy demand is not high.

Therefore, we believe that the design of the route is seriously unreasonable and unscientific in CP2–CP3. And the design at 5–10 km is also absurd. The main reason is that the slope is too large, and the distance with a significant slope is too long^34^.

From Fig. 4, when passing through CP2–CP3, the temperature is lower than the minimum temperature in the previous 21 days, which is also a significant factor leading to the accident. Therefore, it is necessary to design the running route with the local historical temperature and avoid the month with the lowest temperature and the continuous path with a steep slope.

**Figure 6:**
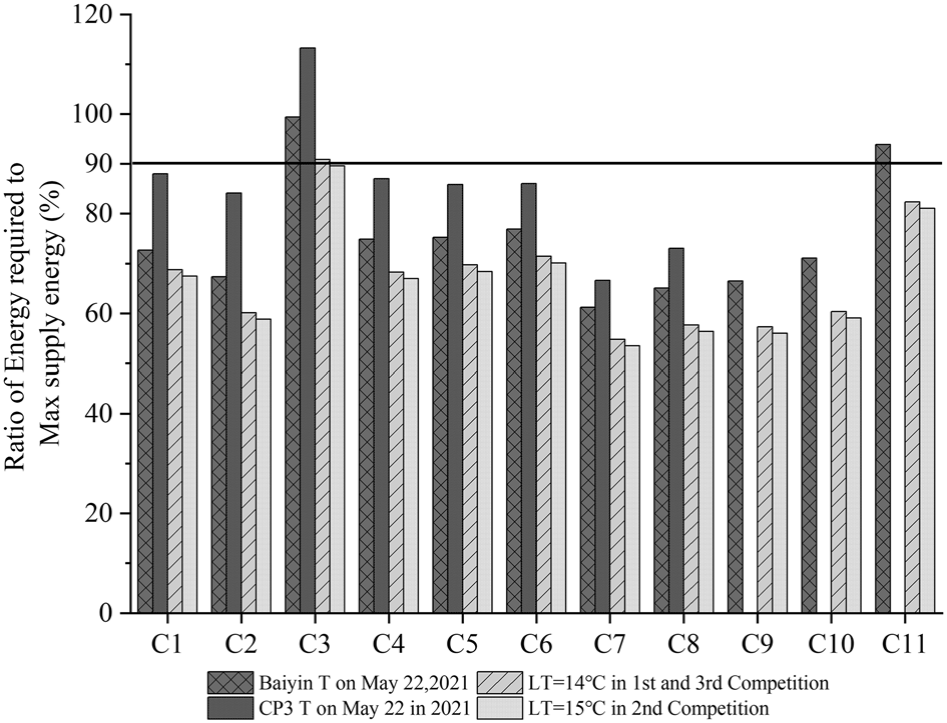
The ratio of the total energy consumed for a marathon to the maximum energy supply T is the temperature, and LT is the lowest temperature.

In the last three marathons, the competition time is May 20, 2018, June 8, 2019, and September 29, 2020. The maximum and minimum temperatures in these three times are 14–27 ℃, 15–29 ℃, 14–20 ℃. Choose the minimum value of temperature for calculation. In each interval, calculate the ratio of the total energy consumed to the maximum supply energy according to Eqs. (1) and (2), shown in Fig. 6.

C1 is from the Start point to CP1 point [Start CP1]. C2: [CP1, CP2]. C3: [CP2, CP3]. C4: [CP3, CP4]. C5: [CP4, SP]. C6: [SP, CP5]. C7: [CP5, CP6]. C8: [CP6, CP7]. C9: [CP7, CP8]. C10: [CP8, CP9].C11: [CP9, End point]. The CP3 T is missing from C9 to C11 from the CP7 to the end. Baiyin T is the temperature of Baiyin. CP3 T is the temperature of CP3 point. The lowest temperature is 14 °C, 15 °C, and 14 °C on the 1st, the competition on May 20, 2018, the second on June 8, 2019, and the third on September 29, 2020. The horizontal black line is 90%.

Section C3 in Fig. 6, that is, from CP2 to CP3, the calculation on the day of the first, second, and third marathon shows that the ratio is close to 90%. Calculate these three results according to the minimum temperatures of 14 °C, 15 °C and 14 °C on the day of the competition. The temperature of the race day is higher than and not constant equal to this minimum temperature. The energy consumption rate in this competition period is less than 90%. At the same time, the span of the minimum and maximum temperature is close to the optimal temperature of 18.6 °C^35^, so the athletes can pass smoothly.

In Fig. 6, in Section C3, according to the temperature at Baiyin meteorological station and CP3 on the day of the fourth competition in 2021, the physical energy consumption of athletes on the race day has been greater than 90%, or even reached 113%, indicating that the physical energy consumption of athletes is too large. This distance is 8.5 km, which takes 1.6 h according to the speed of 1.5 m/s. At this time, the athletes are in danger.

In section C11, in 2021, the physical consumption of athletes has also reached 94%, which is also a dangerous section. In other areas (C1, C2, C4–C10), the physical consumption of athletes does not exceed 90%, and athletes are in a safe state.

The first three were safe from the four Baiyin marathons in 2018, 2019, 2020, and 2021, and the fourth had accidents. Combining Fig. 6 with the above analysis, we conclude that choosing 350 cal/min/kg as Chinese male athletes’ maximum energy supply rate is reasonable under 100% VO_2_max. It is reasonable to take 90% of the energy consumption ratio as Chinese marathon athletes’ maximum energy supply rate 315 cal/min/kg.

## Discussion

The purpose of this study is to prevent the recurrence of such accidents.

The energy consumption of the human body: First, the heat dissipation on the surface of the body is directly related to the outside air temperature. The body heat dissipation is significant when the outside air temperature is low. The second is the path slope. The large slope requires large energy. The third is the moving speed. The speed is large, and the energy required is large. The speed is small, and the energy required is small. The fourth is the wind speed, in Eq. (2). The lost energy is large when the wind speed is large, and the heat loss is slight when the wind speed is negligible.

If completely avoid such accidents, one needs to limit the temperature, slope, moving speed, and wind speed. The above analysis is based on the fact that the long-distance moving speed of ordinary people is 1.5 m/s. The previous analysis has determined that the limit energy supply of personnel is 350 cal/min/kg. According to the calculation of 90%, the maximum energy supply is 315 cal/min/kg to be calculated.

The marathon path is ups and downs with different slopes. The comprehensive slope characterizes the overall slope of the path, which is as follows:

3 $$G_{z} = \frac{{\sum {G_{i} \Delta L_{i} } }}{{\sum {\Delta L_{i} } }} \times 100\% ,$$

where *G*_*z*_ is the comprehensive slope; Δ*L*_*i*_ is the *i*st distance, in the research, Δ*L*_*i*_ = 200 m; *G*_*i*_ is the slope of the *i*st distance, only *G*_*i*_ > 0 is selected for calculation.

The slop of the CP2-CP3 is the largest in the entire marathon route (Fig. 4). This part is chosen to calculate the comprehensive slope according to Eq. (2). The comprehensive slope of the CP2–CP3 is 0.17197. Suppose we determine all the slopes, including the *G*_*i*_ > 0 and *G*_*i*_ < 0, and take − *G*_*i*_ for *G*_*i*_ < 0, the comprehensive slope is 0.21152. It doesn’t take much energy to go downhill, so we don’t take downhill into account. If we take *G*_*i*_ for *G*_*i*_ < 0, the comprehensive slope is 0.132424. This calculation ignores the difficulty of the climb. So we choose 0.17197 to evaluate.

When the temperature is 15 °C, the travel speed is 1.5 m/s, and the wind speed is 0, the required energy is 1546.3 w, which is close to 315 cal/min/kg of the energy supplied by the ordinary human body, and the calculated energy supply rate is 1543.5w.

**Figure 7:**
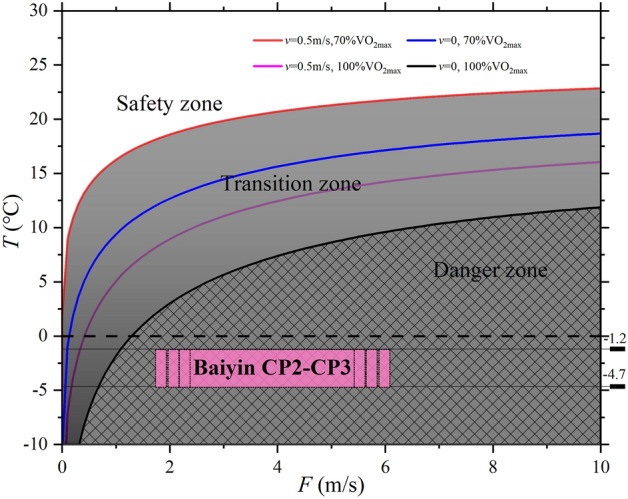
The dangerous risk zones of wind speed and temperature in the CP2–CP3 section of the Baiyin marathon.

According to Eqs. (1), (2), and (3), in the CP2–CP3 section, the slope value is 0.17197, and the temperature and wind speed risk zoning of this section is shown in Fig. 7.

The athlete’s speed is 0, 0.5 m/s, and the maximum energy supply is 1543.5 w, according to 70% and 100% VO_2max_. It is roughly divided into three zones: danger zone, transition zone, and safety zone.

When the Baiyin Marathon was held on May 22, 2021, the temperature range was [− 4.1 ℃, − 1.2 ℃]. Because this paper could not get the wind speed data at that time, according to the photos of the participating remote mobilizers, the wind speed was very large. The wind speed *F* is greater than 5.5–8 m/s, or greater than 14–17 m/s. The dangerous risk zones in CP2–CP3 were shown in “Baiyin CP2–CP3” in Fig. 7. The rectangular area shown in the figure indicates that the site is hazardous, and as the wind speed increases, the danger increases.

In CP2–CP3, marathon runners can make adjustments and actions by assessing their zone based on the temperature and wind speed at that time. Figure 7 has good instructive value and is very actionable. It is convenient for athletes to evaluate whether they can participate in the marathon according to their situation and prepare corresponding equipment.

## Conclusions

‘522’ Baiyin marathon is a typical ‘human life test’ and an ‘energy supply human experiment’. The lessons of the marathon accident can provide a reference for the marathon.

In the first three Baiyin marathons, no accidents occurred. The main reason is that the temperature on the race day is relatively high, which is 14–29 °C. The energy consumption rate of athletes is lower than or temporarily reaches 90%, and the athletes are in a safe state all the time.

An important reason for the accident of the fourth silver marathon in 2021 is the low temperature^15^. In the marathon route design, from CP2 to CP3, the terrain is continuously uphill at a large angle, and the athletes consume much physical energy. From CP2 to CP3, with the most accidents, the athletes’ physical consumption rate is greater than 90% or even 113%, and the athletes are in a dangerous state, resulting in hypothermia. Fatigue leads to insufficient physical strength to resist a sudden low temperature, resulting in hypothermia^36^. Runners used fire to keep warm in a cave-dwelling, shown in Fig. 2d.

There are many reasons^15^ for the 2021 marathon accident, including unscientific route design, unreasonable holding time, etc. These complex factors bring great trouble to the route designer, which is challenging. The method proposed in this paper, which fully considers the slope and temperature, quantitatively calculates the percentage of athletes’ physical consumption, and then designs the route design and evaluation of field marathons through the physical consumption ratio, is a valuable method. The physical energy consumption ratio of 90%, i.e., 315 cal/min/kg, should be taken as the maximum energy supply rate of Chinese marathon athletes and the maximum energy supply rate at the maximum oxygen intake of Chinese male athletes is 350 cal/min/kg.

When designing the marathon route in the future, avoiding continuous uphill with a large slope is necessary. Designers can refer to this literature^34^ for route design. At the same time, the race time should entirely avoid high temperatures and, more importantly, avoid low-temperature times.

At the same time, the dangerous risk zones for the wind speed and temperature of marathon runners are established on the most hazardous path section so that the runners can evaluate whether they can participate in the marathon according to their conditions and prepare corresponding equipment.

Since it is an accident-based analysis, much data is lacking, and life experiments are impossible. The calculation involves a lot of content and is very complex. Nonetheless, such a limited analysis of life is valuable. The guiding significance of the paper is that the organizer needs to calculate the current situation synchronously according to the real-time situation, and broadcast the information in real time.
